# Antithrombotic Therapy in Peripheral Artery Disease: Current Evidence and Future Directions

**DOI:** 10.3390/jcdd10040164

**Published:** 2023-04-10

**Authors:** Mario Enrico Canonico, Raffaele Piccolo, Marisa Avvedimento, Attilio Leone, Salvatore Esposito, Anna Franzone, Giuseppe Giugliano, Giuseppe Gargiulo, Connie N. Hess, Scott D. Berkowitz, Judith Hsia, Plinio Cirillo, Giovanni Esposito, Marc P. Bonaca

**Affiliations:** 1 CPC Clinical Research, Department of Medicine, University of Colorado, Aurora, CO 80045, USA; 2 Department of Advanced Biomedical Sciences, University of Naples Federico II, 80131 Naples, Italy; 3 Quebec Heart and Lung Institute, Laval University, Quebec City, QC G1V0A6, Canada

**Keywords:** peripheral artery disease (PAD), antithrombotic therapy, dual pathway inhibition (DPI), major adverse cardiovascular events (MACE), major adverse limb events (MALE)

## Abstract

Patients with peripheral artery disease (PAD) are at an increased risk of major adverse cardiovascular events, and those with disease in the lower extremities are at risk of major adverse limb events primarily driven by atherothrombosis. Traditionally, PAD refers to diseases of the arteries outside of the coronary circulation, including carotid, visceral and lower extremity peripheral artery disease, and the heterogeneity of PAD patients is represented by different atherothrombotic pathophysiology, clinical features and related antithrombotic strategies. The risk in this diverse population includes systemic risk of cardiovascular events as well as risk related to the diseased territory (e.g., artery to artery embolic stroke for patients with carotid disease, lower extremity artery to artery embolism and atherothrombosis in patients with lower extremity disease). Moreover, until the last decade, clinical data on antithrombotic management of PAD patients have been drawn from subanalyses of randomized clinical trials addressing patients affected by coronary artery disease. The high prevalence and related poor prognosis in PAD patients highlight the pivotal role of tailored antithrombotic therapy in patients affected by cerebrovascular, aortic and lower extremity peripheral artery disease. Thus, the proper assessment of thrombotic and hemorrhagic risk in patients with PAD represents a key clinical challenge that must be met to permit the optimal antithrombotic prescription for the various clinical settings in daily practice. The aim of this updated review is to analyze different features of atherothrombotic disease as well as current evidence of antithrombotic management in asymptomatic and secondary prevention in PAD patients according to each arterial bed.

## 1. Introduction

Peripheral artery disease (PAD) encompasses a variety of non-coronary artery diseases, and its prevalence varies based on screening approaches and clinical features. Recent data reveal a global prevalence of 80 million strokes, the majority (87%) of which are ischemic [1]. Estimated global recent prevalence of abdominal aortic aneurysm (AAA) recognizes a cohort of 35 million patients with AAA, whereas more than 230 million people are affected by lower extremity peripheral artery disease (LEPAD) with increasing prevalence over time due to lack of awareness and consequently underdiagnosis and undertreatment [2,3]. Moreover, epidemiologic data indicate a prevalence of stroke of 3% with 800,000 new/recurrent strokes annually in the United States, with a higher risk in women (20–21%) compared to men (14–17%) for patients aged 55 years or older, and a prevalence of AAA of 0.92% in people aged 30–79 years with a 4:1 ratio for men vs. women [1,3]. The prevalence of LEPAD in men ranges from 6.5% in patients aged 60–69 years to 29.4% in those aged >80 years; in the same age groups, the prevalence of LEPAD in women increases from 5.3% to 24.7% [4,5]. In addition, probably based on genetic and risk factor exposure, LEPAD is more prevalent in black patients than in white patients, whereas prevalence is lowest in Asian and Hispanic patients [4,5]. According to the American College of Cardiology (ACC)/American Heart Association (AHA) and European Society of Cardiology (ESC) guidelines, the PAD definition includes, for each arterial district, a ≥50% stenosis of the extracranial internal carotid artery assessed with the North American Symptomatic Carotid Endarterectomy Trial (NASCET) method, AAA with aortic diameter ≥ 3 cm, and for LEPAD, ankle-brachial index ≤ 0.90, history of claudication, acute limb ischemia (ALI), chronic limb-threatening ischemia (CLTI), amputation for vascular causes or previous lower extremity revascularization (LER) [4,5,6]. Compared with myocardial infarction (MI), PAD shows a more variable clinical presentation, from vague to fatal signs that often lead to delayed diagnosis and treatment [2]. Carotid artery disease manifests a spectrum of different clinical features ranging from asymptomatic cases to hemispheric symptoms such as weakness, numbness, aphasia or face, arm and leg contralateral paresthesia resulting from transient ischemic attack (TIA) or ischemic stroke (IS) [5]. AAA often represents an incidental finding during other imaging tests (e.g., abdominal ultrasound or CT/MRA scan) with usually no specific clinical features, even in patients with more than 5 cm diameter [7]. Life-threatening complications of AAA include aortic rupture with or without previous chronic dissection [7]. LEPAD represents the majority of PAD observed with mild to severe clinical presentations. Claudication represents a mild manifestation of LEPAD, including muscle fatigue, discomfort, cramping or pain triggered by exercise with recovery upon rest [4,5]. ALI represents one of the life-threatening conditions of LEPAD characterized by acute (within 2 weeks) severe limb hypoperfusion with pain, pallor, pulselessness, paresthesia and often paralysis with impaired prognosis in terms of all-cause death and amputation for vascular causes [4,5]. In contrast, CLTI is characterized by chronic (more than 2 week duration) ischemic rest pain, leg nonhealing wound/ulcers or gangrene caused by arterial occlusive disease with poor prognosis [4,5]. Overall, several studies showed an increased risk of major adverse cardiovascular events (MACE), including MI, IS and cardiovascular (CV) death among PAD patients, along with a heightened risk of major adverse limb events (MALE), which is usually defined as severe limb ischemia leading to an intervention or major vascular amputation [4,5]. PAD treatment includes medical therapy, supervised exercise and revascularization (e.g., endovascular, surgical or hybrid) based on anatomical features, patient characteristics and local expertise [4,5,8]. Antithrombotic therapy represents a milestone in PAD management, given that atherosclerosis represents the common pathophysiologic feature in arterial beds [4,5,6,8]. The purpose of this review is to highlight current evidence and future directions on antithrombotic therapy in PAD patients with an overview of the principal trials in this field.

## 2. Role of Atherothrombosis in the Progression and Complications of Non-Coronary Artery Disease

Atherothrombosis represents the common pathophysiologic process in coronary and non-coronary artery disease in patients affected by atherosclerotic damage. PAD involves the same CV risk factors as coronary artery disease (CAD), including arterial hypertension, diabetes mellitus, dyslipidemia, smoking, history of CV disease, chronic kidney disease, life habits, history of radiation therapy, psycho-social and genetic factors [4,5]. These shared factors explain the common finding of polyvascular artery disease, defined by the concomitant presence of relevant atherosclerotic disease in at least two vascular beds [4,5]. However, at variance with CAD and IS, smoking is the risk factor most strongly associated with LEPAD [9]. Finally, differences in atherothrombosis pathophysiology have to be considered among carotid artery disease, abdominal aortic disease and LEPAD.

### 2.1. Carotid Artery Disease

Extracranial carotid atherosclerosis can be readily detected with non-invasive assessment such as high-resolution B-mode carotid ultrasonography (Duplex US), CT and MRI scan also able to detect subclinical atherosclerosis [4,5]. Carotid atherosclerosis leads to 25% of IS associated with disability and impaired prognosis [10]. Stenosis degree is one of the most important risk factors of ipsilateral IS, along with hemodynamic factors. Although hypoperfusion plays a role in the pathogenesis of IS, the majority of stroke events are attributed to embolization from unstable atherosclerotic plaque or carotid artery acute occlusion with thrombus distally detected [10]. As in coronary arteries, vulnerable plaque characteristics include a lipid-rich necrotic core with a thin/ruptured fibrous cap, ulceration and intraplaque hemorrhage (IPH) associated with the presence of inflammatory cells [10]. Carotid stenosis progression is also recently considered a marker of vulnerability, contributing to distal embolization and subsequent TIA [10]. IPH represents one of the plaque progression factors with increased rupture risk and future risk events [10]. Further pathophysiologic findings on vulnerable plaque highlight the role of inflammation in atherosclerosis with intraplaque angiogenesis and hypoxia in cerebral adverse events. Hybrid imaging, such as PET/CT or PET/MRI, can detect plaque rupture features [10]. Some morphologic characteristics, such as ulceration and IPH, are also associated with the occurrence of ischemic events, independently of the degree of stenosis. Recent data from the American Society of Neuroradiology showed that the annualized event rates of ipsilateral stroke in those with IPH are higher than in patients without IPH irrespective of stenosis degree: 9.0% versus 0.7% (<50% stenosis), 18.1% versus 2.1% (50–69% stenosis) and 29.3% versus 1.5% (70–99% stenosis), confirming IPH as an independent predictor of ipsilateral stroke (Hazard Ratio—HR 3.3; 95% confidence interval—CI, 1.4–7.8) [10]. Plaque calcification represents a stabilizing factor in carotid artery disease with less inflammation, neovascularization and IPH and lower likelihood of rupture [10]. Furthermore, several atherosclerosis-related factors such as aging, inflammation and ischemia increase circulating levels and deposition of amyloid-beta (Aβ) in intracranial arteries contributing to different types of dementia with impaired cognitive performance. Moreover, among Aβ peptides, Aβ1-40 was independently associated with impaired vasodilating properties, higher IMT, low ABI, as well as coronary and aorta arterial damage, with a worse prognosis in elderly patients [11]. These findings highlight the interplay between dementia and CVD, particularly driven by diffuse atherosclerosis, although current Aβ pathophysiology and therapeutic options are still uncertain areas.

### 2.2. Abdominal Aortic Disease

Atherosclerosis is frequently associated with abdominal aortic disease, especially in polyvascular disease [7,12]. While the pathophysiologic role of atherosclerosis in medium and small arteries is well-known, the relationship with abdominal aortic disease is incompletely understood. Acute abdominal aortic thrombosis is a fatal and rare condition, and abdominal aortic disease is mostly represented by AAA, which arises as a pathological response to aortic atherosclerosis [12]. In animal models, inflammatory pathways, along with aortic matrix degradation and hemodynamic forces, lead to AAA development [12]. During intraluminal stenosis development, the atherosclerotic process includes compensatory chronic inflammatory changes in the media with extracellular matrix remodeling promoting artery diameter growth leading to the development of an aortic aneurysm [12]. Moreover, aortic media chronic inflammation driven by myo-fibroblast favors aortic false lumen development with chronic aortic dissection origin [7]. To date, the interplay of chronic dissection and aneurysm is not completely understood. Chronic aortic dissection leads to more rapid aortic aneurysm growth than non-dissected aorta [7]. Arterial pressure and relative wall tension drive false lumen propagation in the aortic axis with a high rupture risk, which overcomes the remodeling capability of aneurysmatic artery wall [7]. In addition, partial chronic abdominal aortic thrombosis is a common finding in patients with chronic aortic dissection and/or aneurysm [6]. Often, aortic thrombus shows a multi-layered morphology with dense fibrin and inflammatory cells such as leukocytes and platelet-derived proteins with proteolytic proprieties and increased risk of peripheral embolism [6]. Around 40% of chronic aortic dissection patients require urgent revascularization for aortic rupture and/or branch vessel hypoperfusion [7]. New understandings are evolving from combining 3- and 4-dimensional CT morphology data, MRI flow data, computer simulation of fluid dynamics and the fields of biomechanics and mechanobiology, which may help to better comprehend the physiopathologic key elements leading to false lumen degeneration and aneurysm development and facilitate the development of novel treatments and appropriate timing for them in patients affected by chronic aortic dissection and aneurysm [13,14].

### 2.3. Lower Extremity Peripheral Artery Disease

Atherosclerosis is a common LEPAD feature that can explain symptoms and signs related to different clinical presentations, from claudication to ALI/CLTI [15]. Lower extremity peripheral arteries represent a very diverse arterial bed with several differences and related clinical scenarios between itself. One difference is driven by anatomical factors (e.g., arterial diameter) considering large vessels (e.g., iliac–femoral axis, popliteal artery) and smaller vessels below the knee (BTK) [16]. Consequently, flow characteristics and atherosclerotic complications will be different. Overall, compared to cerebrovascular disease and CAD, the role of atherothrombosis in the progression and complications of LEPAD is less clear and studied. Atherosclerosis causes claudication, which represents the clinical manifestation of significant atherosclerotic stenosis during exercise and relief within 10 min rest. Particularly, symptoms stem from the muscles perfused by the stenosed artery [5,7]. Similar to chronic CAD, claudication represents the chronic manifestation of LEPAD, with management depending mostly on CV risk factor and physical exercise management [5,7]. Considering atherothrombotic complications of LEPAD, approximately 10% of patients with claudication develop CLTI within 5 years, contributing to poor prognosis, including 1-year rates of mortality of 25% and 1-year rates of amputation of 30% [15]. The main difference from CAD atherothrombosis is the occurrence of thrombotic events even in the absence of significant atherosclerotic disease. Histopathological analysis on LEPAD presenting with CLTI shows that thrombotic occlusion in the BTK district is the main cause of disease even in patients without significant atherosclerosis, while significant atherosclerotic lesions were more often detected in the femoral-popliteal artery [16]. On the contrary, ALI and related thrombus often occur in patients with both significant and non-significant atherosclerosis, while small vessel obliteration is driven by media calcification, intimal fibrosis and superimposed cholesterol emboli [16]. Similar to acute MI, ALI is characterized by a sudden decrease in limb perfusion that often results in tissue loss and requires early intervention. However, in contrast to atherothrombotic acute coronary events, ALI in patients with PAD is driven not only by atherothrombosis but also by emboli from the heart and proximal vessels and graft occlusion in patients with previous lower extremity revascularization (LER) [17]. Several pieces of evidence support the thromboembolic origin of CLTI/ALI-affected popliteal and BTK artery rather than stenotic atherosclerotic disease. The embolic source is often an aorto-iliac-femoral atherosclerotic plaque with subsequent lumen obliteration of a distal smaller artery [16]. The main differences between ALI and CTLI are represented by the duration of symptoms (less vs. more than two weeks), clinical presentation (acute vs. chronic), presence of collateral arteries in CTLI and timing of revascularization (urgent in order to address the high risk of amputation vs. non-urgent in order to minimize tissue loss) [18].

## 3. Approaches to Antithrombotic Therapy in Peripheral Artery Disease

**Figure 1:**
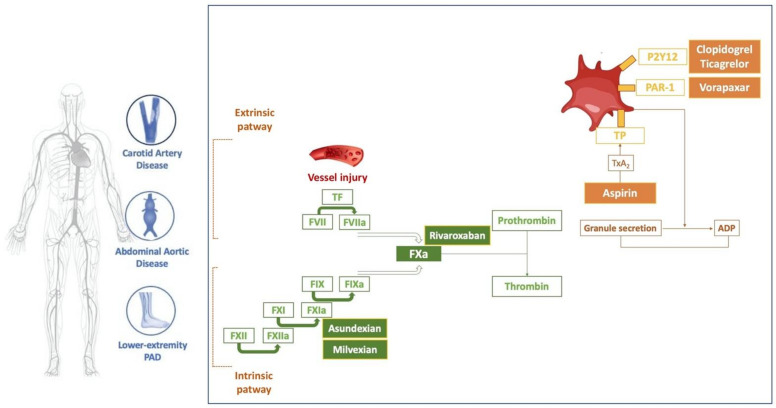
Pharmacodynamic targets of antithrombotic drugs in peripheral artery disease by thrombotic pathway. TF = tissue factor, TxA_2_ = Thromboxane A2, TP = thromboxane prostanoid, PAR-1 = protease-activated receptor-1, ADP = adenosine diphosphate.

Guideline-directed medical therapy (GDMT) includes antithrombotic therapy as one of the cornerstones of multidimensional management, which includes structured exercise and lifestyle modification in order to reduce MACE and MALE [4,5,6]. The well-recognized role of atherosclerosis and its related complications in PAD patients explains the need for antithrombotic therapy in each arterial bed, even in asymptomatic patients [4,5]. Antithrombotic management in PAD represents a challenge due to different evidence for each arterial bed, symptoms assessment and personal expertise. While the importance of antithrombotic therapy in CAD has been well-recognized over the past decades and includes dedicated randomized clinical trials (RCTs), evidence supporting antithrombotic therapy in PAD has been based, until recently, on subgroup analyses of coronary artery disease trials, often with slim and conflicting data. In the last years, a new antithrombotic strategy emerged in PAD research that combines an anticoagulant with standard antiplatelet therapy. The recognized role of embolic source of many cases of LEPAD promoted a new target therapy called dual pathway inhibition (DPI) to inhibit thrombus formation via dual pathways: platelet activation and thrombin generation [19]. In addition, thrombo-hemorrhagic risk has to be assessed in order to choose the right antithrombotic regimen. Due to concomitant diseases, PAD patients have a high bleeding risk compared to the CAD population, but there is less evidence on which to develop a bleeding score risk assessment. To date, Thrombolysis in Myocardial Infarction (TIMI) and the International Society on Thrombosis and Hemostasis (ISTH) bleeding are the most common safety outcomes assessed in PAD trials [20]. Pharmacodynamic targets of antithrombotic drugs in peripheral artery disease by thrombotic pathway are summarized in Figure 1.

### 3.1. Carotid Artery Disease

#### 3.1.1. Asymptomatic Patients

GDMT includes antithrombotic therapy in carotid artery disease in the presence of a ≥50% stenosis since no RCT has assessed antithrombotic therapy in non-significant carotid stenosis [5,19]. In asymptomatic patients with significant carotid stenosis, GDMT recommends single antiplatelet therapy (SAPT) either with aspirin (75–100 mg) or clopidogrel (75 mg) (Class IIa) for primary prevention of MACE if bleeding risk is low [5,21]. The use of aspirin in overall PAD patients was assessed in AntiThrombotic Trialists (ATT) meta-analysis, including six primary prevention RCTs assessing different doses of aspirin as well as other antiplatelet agents such as picotamide. In primary prevention RCTs, aspirin reduced serious vascular events (including MI, stroke and vascular death) by 12% (HR 0.88; 95% CI 0.82–0.94 without benefit on CAD death, HR 0.95; 95% CI 0.78–1.15 or stroke death, HR 1.21; 95% CI 0.84–1.74), associated with an increase of hemorrhagic stroke HR, 1.32; 95% CI 1.00–1.75 and major extracranial bleeding (HR 1.54; 95% CI 1.30–1.82) [22]. These results confirm the uncertain net benefit of aspirin in PAD primary prevention in the absence of concomitant diseases. Recently, an analysis on stroke risk in Cardiovascular Outcomes for People Using Anticoagulation Strategies (COMPASS) trial, comparing rivaroxaban 2.5 mg twice daily plus aspirin vs. rivaroxaban 5 mg twice daily vs. aspirin in stable CAD or PAD patients, proved the efficacy of rivaroxaban 2.5 mg plus aspirin with a 53% relative reduction in the risk of ischemic/unknown stroke in high-risk patients without a history of stroke (HR, 0.57; 95% CI 0.39–0.84), with no significant increase in the risk of hemorrhagic stroke for rivaroxaban plus aspirin vs. aspirin alone (HR, 1.76; 95% CI 0.59–5.24) [23]. Therefore, a low dose of rivaroxaban plus aspirin could represent a new antithrombotic regimen for polyvascular disease patients with CAD and/or LEPAD without prior history of stroke, especially among those without high bleeding risk features. However, appropriately designed trials are needed to share light in this field.

#### 3.1.2. Secondary Prevention

**Figure 2:**
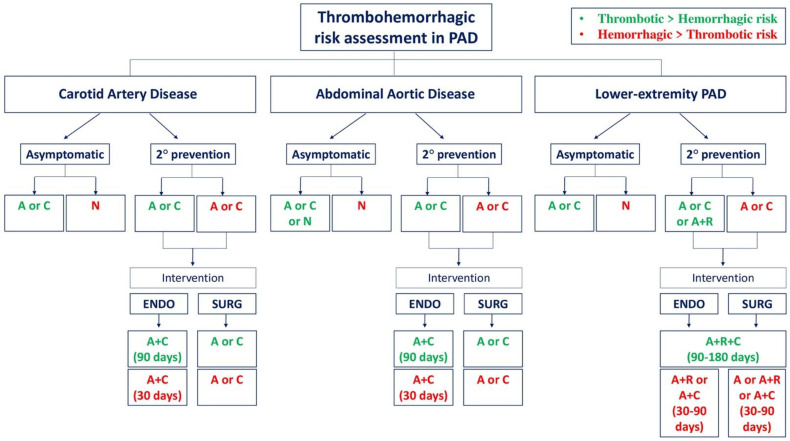
Proposed algorithm for antithrombotic management in patients with PAD asymptomatic or in secondary prevention. A = low-dose aspirin, C = clopidogrel 75 mg/day, N = no medical therapy, R = rivaroxaban 2.5 mg/twice daily, ENDO = endovascular revascularization, SURG = surgical revascularization.

Antithrombotic therapy is recommended in patients with symptomatic carotid artery disease to prevent recurrent cerebrovascular events [8,19]. GDMT recommends lifelong SAPT with aspirin or clopidogrel in patients with prior IS or TIA due to large artery disease [5,8]. SAPT showed a better safety profile in major bleeding outcomes compared to oral anticoagulation. Data from the ATT meta-analysis among 16 secondary prevention RCTs, including 10 with previous stroke/TIA, highlighted the benefit of aspirin on major coronary events (HR 0.80; 95% CI 0.73–0.88) as well as IS (HR 0.78; 95% CI 0.61–0.99) and serious vascular events (HR 0.81; 95% CI 0.75–0.87), with an increase of major extracranial bleeding (HR 2.69; 95% CI 1.25–5.76) but not for rates of hemorrhagic stroke (HR 1.67; 95% CI 0.97–2.90) [22]. Different RCTs compared oral anticoagulation vs. SAPT in PAD patients (Table 1). In the European/Australasian Stroke Prevention in Reversible Ischemia Trial (ESPRIT), oral anticoagulation (either phenprocoumon, warfarin or acenocoumarol) was compared with aspirin in patients with recent (within 6 months) TIA or minor stroke. Overall, there was no significant difference between the two groups for recurrent CV events (HR 1.02; 95% CI 0.77–1.35). However, an excess of major bleeding among patients randomized to oral anticoagulation was observed (HR 2.56; 95% CI 1.48–4.43) [24]. The Warfarin Antiplatelet Vascular Evaluation (WAVE) compared a combination therapy with an antiplatelet agent (either aspirin, ticlopidine or clopidogrel) plus oral anticoagulant agent (either warfarin or acenocoumarol) vs. antiplatelet therapy alone in PAD patients. No differences were detected in the primary efficacy outcome of MACE (RR 0.92; 95% CI 0.73–1.16), although the risk of life-threatening bleeding was three-fold higher in the combination therapy arm (RR 3.41; 95% CI 1.84–6.35) [25]. Hence, unless indicated for other clinical circumstances, anticoagulation therapy is not recommended for secondary prevention after TIA/stroke [5,8]. Other antiplatelet drugs, such as P2Y12 receptor inhibitors, have been assessed in secondary IS prevention. In the Clopidogrel and Aspirin for Reduction of Emboli in Symptomatic Carotid Stenosis (CARESS) trial, dual antiplatelet therapy (DAPT) with aspirin and clopidogrel was compared to aspirin among patients with recently symptomatic ≥ 50% carotid stenosis assessing microembolic signals (MES) by transcranial Doppler (TCD). DAPT was associated with a relative reduction of 40% in the risk of asymptomatic embolization and stroke (relative risk reduction—RRR 39.8%; 95% CI 13.8–58.0) on day 7 with no significant difference in bleeding adverse events among DAPT (3.9%) vs. aspirin alone (1.8%) group [26]. Similarly, in the clopidogrel plus aspirin versus aspirin alone for reducing embolization in patients with acute symptomatic cerebral or carotid artery stenosis (CLAIR) trial, DAPT with aspirin and clopidogrel compared with aspirin alone was associated with similar risk reduction (about 42%) of MES in patients with acute IS or TIA (RRR 42.4%; 95% CI 4.6–65.2) with only two minor bleeding events in the DAPT group [27]. The Clopidogrel in High-Risk Patients with Acute Nondisabling Cerebrovascular Events (CHANCE) trial assessed a 3-month strategy of DAPT with aspirin plus clopidogrel vs. aspirin alone among patients with acute high-risk TIA or minor ischemic stroke. During 90 days of follow-up, DAPT reduced the occurrence of stroke (ischemic or hemorrhagic) by 32% compared to aspirin alone (HR 0.68; 95% CI 0.57–0.81) with a non-significant increased rate of any bleeding events in DAPT (2.3%) vs. aspirin group (1.6%) (HR 1.41; 95% CI 0.95–2.10) [28]. Like the CHANCE trial, the Platelet-Oriented Inhibition in New TIA and Minor Ischemic Stroke (POINT) trial assessed DAPT with aspirin and clopidogrel vs. aspirin alone in patients affected by minor IS or high-risk TIA for 90 days. DAPT reduced the composite primary efficacy outcome of major ischemic events defined as IS, MI and death due to ischemic vascular events (HR 0.75, 95% CI 0.59–0.95), which was counterbalanced by an increased risk of major bleeding in DAPT arm of HR (2.32, 95% CI 1.10–4.87) [29]. A pooled analysis of the CHANCE and POINT trials showed a reduced risk of new stroke with DAPT (HR 0.69; 95% CI 0.60–80), with a non-significantly higher risk of major bleeding (HR 1.67, 95% CI 0.93–2.99) [30]. A risk benefit-analysis of the two trials showed that the recurrent ischemic events with DAPT are mainly prevented within the first 2 weeks after randomization, whereas the risk of major bleeding was small and constant throughout the follow-up. In view of these findings, the AHA/ASA guidelines recommend DAPT with clopidogrel and aspirin up to 3 months after a minor stroke [8]. In analogy with the issues related to clopidogrel use in the field of CAD (delayed onset of action, large interindividual variability and irreversibility of inhibitory action), data from the CHANCE trial showed that the benefit of a clopidogrel-based DAPT was essentially confined to extensive clopidogrel metabolizer phenotype, whereas there was no benefit with DAPT in poor or intermediate clopidogrel metabolizers [31]. Cilostazol is another drug assessed in the secondary prevention of IS. Cilostazol is a selective inhibitor of phosphodiesterase type 3, which increases the cyclic adenosine monophosphate (cAMP) levels that lead to inhibition of platelet aggregation. “Dual Antiplatelet therapy using Cilostazol for Secondary Prevention in Patients with high-risk ischemic stroke in Japan”, a multicenter RCT, assessed the safety and efficacy of cilostazol to prevent stroke recurrence in a DAPT strategy with either aspirin or clopidogrel vs. SAPT with aspirin or clopidogrel. DAPT with cilostazol reduced IS recurrence (3%) vs. SAPT (7%) (HR 0.49; 95% CI 0.31–0.76) with no differences in severe or life-threatening bleeding among study groups (HR 0.66; 95% CI 0.27–1.60) [32]. However, the RCT enrolled 47% of the planned sample size (n = 4000) and included only Japanese people. Moreover, there were a very limited number of patients (n = 93) with primary efficacy outcomes that could probably reduce the statistical accuracy [32]. AHA/ASA guidelines recommend cilostazol with a class of recommendation 2b in IS secondary prevention with aspirin or clopidogrel due to limited evidence and known side effects such as headache, palpitations and tachycardia. In addition, Cilostazol is contraindicated in patients affected by heart failure (HF) treated by phosphodiesterase 3 inhibitors [8]. The efficacy and safety of a different antiplatelet strategy after IS/TIA were also assessed with newer P2Y_12_ receptor inhibitors. The benefit of ticagrelor compared to aspirin in patients with acute cerebral ischemia (non-severe IS or high-risk TIA) was assessed among 13,199 patients enrolled in the Acute Stroke or Transient Ischemic Attack Treated with Aspirin or Ticagrelor and Patient Outcomes (SOCRATES) trial. Compared with aspirin, ticagrelor monotherapy (90 mg twice daily) was not superior to aspirin with respect to the primary outcome of stroke, MI or death at 90 days (HR 0.89; 95% CI 0.87–1.01). No differences were detected in major bleeding (according to PLATO classification) comparing the two study groups (HR 0.83; 95% CI 0.52–1.34) [33]. However, in a sub-analysis of the SOCRATES trial in which the primary outcome data were stratified by randomization arm and prior use of aspirin within 7 days before randomization, the benefit of ticagrelor was present among patients with prior aspirin use (HR 0.76; 95% CI 0.61–0.95) [34]. Recently, DAPT, including the more potent P2Y12 receptor inhibitor (ticagrelor 90 mg twice daily) on top of background therapy, was investigated in The Acute Stroke or Transient Ischemic Attack Treated with Ticagrelor and ASA (acetylsalicylic acid) for Prevention of Stroke and Death (THALES) trial, which enrolled patients with no more than moderate and non-cardioembolic IS. DAPT with ticagrelor reduced the risk of IS or death within 30 days (HR 0.83; 95% CI 0.71–0.96) with no differences in disability. However, severe bleeding occurred more frequently in patients randomized to the experimental arm according to the Global Use of Strategies to Open Occluded Coronary Arteries (GUSTO) classification (HR 3.99, 95% CI 1.74–9.14) [35]. Considering the thromboembolic component of IS, recent data from a post-hoc analysis on DPI comes from RCT. In particular, the analysis of stroke outcomes in the COMPASS trial among patients with prior stroke showed a benefit of low-dose rivaroxaban plus aspirin compared with aspirin alone in MACE prevention (HR 0.57; 95% CI 0.34–0.96) without differences in ISTH major bleeding (HR 1.06; 95% CI 0.72–1.56) [23]. Nevertheless, these results cannot be extrapolated to the early phase after IS. In the early phase of a cerebrovascular event, DAPT represents the antithrombotic strategy in order to minimize the risk of asymptomatic cerebral embolization and stroke [19]. Antithrombotic therapy in secondary prevention of carotid artery disease was also assessed after CAS/CEA revascularization. Data derived from two small RCTs showed benefit from the DAPT regimen vs. SAPT after CAS in reducing cerebrovascular events. “The Benefits of Combined Anti-platelet Treatment in Carotid Artery Stenting” RCT compared DAPT with aspirin plus clopidogrel vs. aspirin plus 24 h of heparin in a cohort of patients undergoing CAS. At 30 days of follow-up, neurological complications, including all amaurosis fugax, TIA and all stroke, occurred in 0% of the DAPT group and in 25% of heparin group (*p* = 0.02) without any difference in major bleeding or groin complication (9% in DAPT group vs. 17% in heparin group, *p* = NS) [36]. Similar findings were noted in “Dual Antiplatelet Regime Versus Acetyl-acetic Acid for Carotid Artery Stenting” RCT in patients after CAS assessing a DAPT regimen (325 mg of aspirin plus 250 mg of ticlopidine) vs. 325 mg of aspirin plus 24 h of heparin as control group. After 30 days, DAPT significantly reduced minor IS/TIA (2% vs. 16%, *p* < 0.05) without any major bleeding in either group, and no difference in groin complications was observed (2% in DAPT vs. 4% in control group, *p* = NS) [37]. The optimal SAPT strategy after CEA was tested in low-dose and high-dose acetylsalicylic acid for patients undergoing carotid endarterectomy: a randomized controlled trial (ACE RCT) where different doses of aspirin (i.e., 81–325 mg vs. 650–1300 mg) were compared at 3 months after CEA. The occurrence of IS was lower in low-dose aspirin (3.2% vs. 6.9%), while hemorrhagic stroke was numerically more frequent in the high-dose aspirin group (RR 1.68, 95% CI 0.77–3.68) [38]. Moreover, after CEA, SAPT management is recommended over a DAPT regimen in view of a lack of benefit in fatal stroke prevention and heightened risk of major bleeding with DAPT, as shown in a systematic metanalysis involving three RCTs and seven observational studies with DAPT vs. SAPT risk difference (RD) in major bleeding 0.00; 95% CI 0.00–0.01 and neck hematoma (RD 0.04, 95% CI 0.01–0.06) [39]. The need to reduce thrombotic adverse events without increasing bleeding risk drives the development of newer antithrombotic drugs with a different target in atherosclerotic cardiovascular disease (ASCVD), including carotid artery disease for non-cardioembolic IS. Considering the key role of factor XI (FXI) in pathological thrombosis as it amplifies thrombin generation, new data provide the first clinical benefits of factor XI inhibitors [40]. Moreover, patients with FXI deficiency are known to have a lower risk of IS, while those with high FXI levels have an increased risk of recurrent IS [40]. The Factor XIa inhibition with asundexian after acute non-cardioembolic ischemic stroke (PACIFIC-Stroke) is a phase 2b RCT, the first study to report on the efficacy and safety of factor Xia inhibition in secondary prevention of non-cardioembolic IS. Patients with minor-moderate IS were randomized in a 1:1:1:1 study to receive either asundexian 10 mg, 20 mg, or 50 mg or placebo, in addition to SAPT, according to a local investigator. After 26 weeks from randomization, no differences were observed in primary efficacy outcome (i.e., symptomatic recurrent IS and incident covert brain infarct detected by MRI) among each asundexian arm vs. placebo: HR 0.99; 95% CI 0.79–1.24 for 10 mg, HR 1.15; 95% CI 0.93–1.43 for 20 mg and HR 1.06; 95% CI 0.85–1.32 for 50 mg. Considering the primary safety composite outcome of ISTH major and clinically relevant non-major bleeding, no significant differences were found among each asundexian arm and even considering all doses vs. placebo (HR 1.57, 95% CI 0.91–2.71) [41]. Despite the PACIFIC-stroke trial not finding a significant difference in the primary efficacy endpoint, in a post-hoc analysis, asundexian 50 mg reduced recurrent IS and TIA in patients with known atherosclerosis. This reinforced the rationale for assessing this hypothesis in a larger phase 3 RCT. Another XIa inhibitor, milvexian, was more recently tested in the Antithrombotic treatment with factor XIa inhibition to Optimize Management of Acute Thromboembolic events for Secondary Stroke Prevention (AXIOMATIC-SSP) phase 2 RCT in patients with acute IS or high-risk TIA compared to matched placebo on top of DAPT with aspirin and clopidogrel until day 21 from randomization followed by aspirin alone. The primary efficacy endpoint included incident IS during the treatment period or new covert brain infarction detected by the comparison of 90-day and baseline MRIs. Although the full results are not yet available at the time of this writing, preliminary data from the ESC 2022 Congress showed no difference between milvexian and placebo in the primary efficacy endpoint at 90 days. As it relates to the safety endpoint (Bleeding Academic Research Consortium—BARC classification), milvexian did not increase adverse events: milvexian 25 mg daily vs. 25 mg BID vs. 50 mg BID vs. 100 mg BID vs. 200 mg BID vs. placebo, was: 10.8% vs. 8.6% vs. 12.3% vs. 13.1% vs. 10.2% vs. 7.9% (*p* > 0.05) [42]. To date, GDMT and the recent ESC consensus document recommend at least DAPT for 1-month with aspirin and clopidogrel after CAS and SAPT after CEA (class I) [4,5,19]. An operative proposal algorithm for antithrombotic management of carotid artery disease according to asymptomatic or secondary prevention patients is depicted in Figure 2.

### 3.2. Abdominal Aortic Disease

#### 3.2.1. Asymptomatic Patients

GDMT does not recommend SAPT in asymptomatic abdominal aortic atherosclerosis overall [6,19]. Despite the common finding of abdominal aortic atherosclerosis in middle-aged patients, SAPT therapy should be avoided unless complex and high-risk plaque such as atheroma dimension >3 mm or >2 mm with mobile/ulcerated features, which confer an increased risk of MACE is observed [19]. Considering the relationship between intra-luminal thrombus and AAA, a hypothesis of antithrombotic treatment efficacy on the growth rate of AAA was assessed in The Efficacy of Ticagrelor on Abdominal Aortic Aneurysm (AAA) Expansion (TicAAA), where ticagrelor was compared with placebo for reducing AAA over 12 months. No differences were found in AAA volume increase with ticagrelor vs. placebo as assessed by MRI (HR 1.013; 95% CI 0.993–1.034) and in diameter change assessed by US for ticagrelor (2.3 mm) vs. placebo (2.2 mm), *p* = 0.778 [43]. Moreover, patients in the ticagrelor group showed an increased rate of bleeding events (33% vs. 11%, *p* = 0.002) (Table 1) [43]. A recent review and metanalysis of seven studies on antiplatelet therapy (including aspirin) in AAA confirms the absence of benefit on aneurysm growth by antiplatelet therapy with an overall standardized mean difference (SMD) of −0.36 mm/year, 95% CI −0.75–0.02 [53]. In addition, compared to placebo, aspirin use in AAA was not associated with reduced all-cause mortality (HR 0.91; 95% CI 0.75–1.11) or abdominal aortic rupture events (HR 0.98; 95% CI 0.37–2.59) [53]. Given the higher bleeding risk, DAPT or oral anticoagulation are not indicated for primary prevention of abdominal aortic aneurysm [19].

#### 3.2.2. Secondary Prevention

After a peripheral embolic event from abdominal aortic plaque, GDMT recommends SAPT (aspirin or clopidogrel), whereas little evidence is available for DAPT in this clinical scenario [6,19]. Due to the higher risk of MACE in patients affected by AAA, unless contraindicated, SAPT is a reasonable option [19]. As mentioned before, intraluminal thrombus is a common finding in patients with AAA. Parenteral administration of factor Xa/II inhibitors in experimental aortic aneurysms and atherosclerosis were assessed in an animal model. Reduction in the severity of aortic aneurism and atherosclerosis was detected in mice treated with enoxaparin or fondaparinux [54]. Even in the setting of AAA complications (e.g., aortic dissection), SAPT should not be withdrawn in order to prevent thrombosis origin and propagation [19]. Data on antithrombotic therapy after AAA repair are still limited and mostly of an observational nature. In patients undergoing intervention (either EVAR or surgical) for AAA, pooled data from observational studies showed no effect on all-cause mortality with antithrombotics compared to placebo/no treatment (HR 1.00; 95% CI 0.81–1.22) [53]. However, studies comparing aspirin vs. placebo/no treatment show a reduction in all-cause mortality in the aspirin arm (HR 0.78; 95% CI 0.68–0.89) with apparent early endoleak risk (<30 days) increase in patients on antithrombotics treatment (HR 1.63; 95% CI 1.17–2.27) [53]. In a small prospective cohort of AAA patients undergoing EVAR, a DAPT strategy showed low complication rates (i.e., 30 day-mortality and endoleak) [55]. During perioperative management of isolated AAA by endovascular treatment, a short-term DAPT (1–3 months) may be indicated [56]. Moreover, DAPT strategy after EVAR showed a good efficacy and safety profile in an observational cohort of patients who underwent recent percutaneous coronary intervention (PCI) compared to SAPT considering BARC major bleeding (0% vs. 1.1%, *p* = NS), endoleak (0% vs. 3.4%, *p* = NS) and MI (2.4% vs. 0%, *p* = NS) [57]. Very limited experience with more potent DAPT (e.g., with ticagrelor or prasugrel) exhibited an increased bleeding risk after AAA repair, irrespective of intervention type [56]. Moreover, recent data on the Safety of Chronic Anticoagulation Therapy After Endovascular Abdominal Aneurysm Repair registry underlined the use of anticoagulation drugs (i.e., vitamin K antagonists/heparin) is independently associated with an increased risk of endoleak (HR 1.6; 95% CI 1.23–2.07) and reintervention (HR 1.8; 95% CI: 1.31–2.48) compared to patients with SAPT [58]. An operative proposal algorithm for the antithrombotic management of abdominal aortic disease according to asymptomatic or secondary prevention patients is represented in Figure 2.

### 3.3. Lower Extremity Peripheral Artery Disease

#### 3.3.1. Asymptomatic Patients

Antithrombotic management recommendations in patients with isolated asymptomatic LEPAD are conflicting. ESC guidelines and a recent ESC position paper do not recommend SAPT in asymptomatic LEPAD, whereas ACA/AHC guidelines acknowledge SAPT as a reasonable option in asymptomatic patients with abnormal ABI (≤0.90) to reduce MI, IS and vascular death risk [4,5,19]. Two RCTs assessed antiplatelet therapy as primary prevention in LEPAD patients. In the prevention of progression of arterial disease and diabetes (POPADAD) trial, aspirin did not show benefit vs. placebo for MACE or major amputation (HR 0.98; 95% CI 0.76–1.26 in primary prevention in diabetes patients with ABI ≤ 0.99). No differences in gastrointestinal bleeding were detected (HR 0.90; 95% CI 0.53–1.52) [44]. The Aspirin for Prevention of Cardiovascular Events in a General Population Screened for a Low Ankle Brachial Index (AAA) trial assessed the role of aspirin in a general population with impaired ABI ≤ 0.95. Considering fatal or non-fatal coronary events or stroke or revascularization, no statistically significant differences were found between aspirin vs. placebo (HR 1.03; 95% CI 0.84–1.27) but there was increased major bleeding in the aspirin arm (HR, 1.71; 95% CI 0.99–2.97) [45]. SAPT seems a reasonable option in primary prevention in patients with impaired ABI with low bleeding risk [4,5,19].

#### 3.3.2. Secondary Prevention

GDMT supports antithrombotic therapy for the secondary prevention of LEPAD with SAPT (aspirin or clopidogrel) with a class I recommendation [4,5,59]. ACC/AHA guidelines endorse DAPT (aspirin plus clopidogrel) and vorapaxar added on top of DAPT in class IIb recommendation in symptomatic PAD [4]. Moreover, a recent ESC consensus paper endorses aspirin plus rivaroxaban 2.5 mg with or without clopidogrel after LER in patients without high bleeding risk [19]. Several new lines of evidence are available considering different clinical scenarios (e.g., medical therapy management alone vs. LER) balancing thrombo-hemorrhagic risk (Table 1) [19]. Historically, data on LEPAD antithrombotic therapy were extracted by subgroup analysis of non-dedicated RCTs on CAD patients [15,60]. More than 25 years ago, subgroup analysis on clopidogrel versus aspirin in patients at risk of ischemic events (CAPRIE), including 6452 symptomatic PAD patients, highlighted MACE prevention in clopidogrel-allocated subjects over aspirin (HR 0.78; 95% CI 0.65–0.93). Moreover, in the overall cohort of the study, clopidogrel showed a favorable gastrointestinal bleeding profile compared to aspirin (1.99% vs. 2.66%, *p* < 0.05) with no differences in intracranial hemorrhage (0.35% vs. 0.49%, *p* = NS) [61]. In the LEPAD patients subgroup analysis of The Clopidogrel for High Atherothrombotic Risk and Ischemic Stabilization, Management and Avoidance (CHARISMA) trial on the efficacy and safety of clopidogrel plus aspirin as compared with aspirin alone in patients at high risk for a cardiovascular event, no benefit was observed for DAPT over SAPT considering MACE (HR 0.85; 95% CI 0.66–1.08) [19]. However, subsequent analyses from the CHARISMA trial showed a favorable effect of DAPT vs. SAPT with aspirin in high-risk patients with prior MI (HR 0.78; 95% CI 0.61–0.98), prior IS (HR 0.78; 95% CI 0.62–0.97) and also with a history of LEPAD (HR 0.83; 95% CI 0.72–0.95). The overall rates of moderate-severe/fatal bleeding did not differ between the groups, whereas minor bleeding increased with DAPT vs. SAPT (HR 1.99; 95% CI 1.69–2.34) [62]. As in carotid artery disease, the feasibility of oral anticoagulation on top of SAPT vs. SAPT alone was assessed in the WAVE study, where LEPAD patients made up around 80% of the overall cohort. No differences were found in the primary efficacy outcome of MACE (RR 0.92; 95% CI 0.73–1.16), but there was a significant increase in life-threatening bleeding with combination therapy (RR 3.41; 95% CI 1.84–6.35) [25]. Unless prescribed for another indication, oral anticoagulation on top of SAPT in symptomatic LEPAD patients is not recommended due to increased bleeding risk [4,5,19]. A DAPT regimen of aspirin and ticagrelor for secondary prevention among PAD patients was evaluated in the subanalysis of the Prevention of Cardiovascular Events in Patients With Prior Heart Attack Using Ticagrelor Compared to Placebo on a Background of Aspirin—Thrombolysis In Myocardial Infarction 54 (PEGASUS-TIMI 54) trial. This RCT assessed the efficacy of ticagrelor 90 mg twice daily, ticagrelor 60 mg twice daily or placebo on top of aspirin in secondary prevention 1 to 3 years after MI. In the LEPAD subgroup ticagrelor 60 mg reduced CV mortality compared to placebo (HR 0.47; 95% CI 0.25–0.86). Considering pooled doses, ticagrelor reduced ALI (HR 0.56; 95% CI 0.23–1.37) and peripheral revascularization for limb ischemia (HR 0.63; 95% CI 0.43–0.93). However, the pooled ticagrelor dose group had a numerical increase of TIMI major bleeding (HR 1.32; 95% CI 0.41–4.29) [63]. DAPT with ticagrelor was also assessed in The Effect of Ticagrelor on Health Outcomes in Diabetes Mellitus Patients Intervention Study (THEMIS) RCT, which enrolled diabetic patients without prior MI or IS to ticagrelor 90 mg twice daily plus aspirin or placebo plus aspirin. Ticagrelor reduced MACE (HR 0.90; 95% CI 0.81–0.99) and MALE (HR 0.45; 95% CI 0.23–0.86) counterbalanced by increased TIMI major bleeding (HR 2.32; 95% CI 1.82–2.94) [64]. In the THEMIS-PAD substudy, DAPT significantly reduced limb events (defined as peripheral revascularization, ALI, major amputation) by 1.3% vs. 1.6% with SAPT (*p* = 0.022) counterbalanced by increased TIMI major bleeding of 2.0% vs. 1.1%, *p* < 0.0001. The overall benefit was greater among PAD patients compared to those without [64]. Ticagrelor was also compared to clopidogrel in the Examining Use of Ticagrelor in Peripheral Artery Disease (EUCLID) trial that investigated the efficacy of ticagrelor, compared to clopidogrel, for reduction of MACE in patients with symptomatic LEPAD. No difference was found for MACE (HR 1.02; 95% CI 0.92–1.13) or major bleeding (HR 1.10, 95% CI 0.84–1.43) between groups, highlighting ticagrelor as not superior to clopidogrel in symptomatic LEPAD patients [46]. Unless for another indication, DAPT therapy is not indicated vs. SAPT in symptomatic LEPAD patients managed with medical therapy alone. Another therapeutic target assessed in the last years is represented by thrombin inhibition on top of low-dose aspirin therapy in DPI. Vorapaxar, a competitive and selective antagonist of thrombin receptor PAR-1, was assessed in Thrombin Receptor Antagonist in Secondary Prevention of Atherothrombotic Ischemic Events–Thrombolysis in Myocardial Infarction 50 (TRA 2°P–TIMI 50) trial, where vorapaxar 2.5 mg daily was compared against placebo on top of standard therapy (e.g., aspirin or clopidogrel) in the secondary prevention of patients with a history of MI, IS or PAD [65]. Overall, vorapaxar reduced the risk of CV death, MI, stroke or recurrent ischemia leading to revascularization (HR 0.88; 95% CI 0.82–0.95) with an increased rate of moderate–severe bleeding than placebo (HR 1.66; 95% CI 1.43–1.93), including intracranial hemorrhage [65]. Subsequent analysis of the PAD population showed greater benefits of vorapaxar in limb events with a 42% reduction in hospitalization for ALI (HR 0.58; 95% CI 0.39–0.86) and a significant reduction in peripheral artery revascularization (HR 0.84; 95% CI 0.73–0.97) although evidence of increased GUSTO moderate–severe bleeding in the vorapaxar arm (HR 1.62; 95% CI 1.21–2.18) [66]. Further data among patients with PAD confirmed the clinical benefits of vorapaxar on MACE in patients with concomitant CAD (HR 0.85; 95% CI 0.73–0.99) and MALE in those with prior LER (HR 0.67; 95% CI 0.49–0.91) accompanied by an increase of ISTH major bleeding by 39% (HR 1.39; 95% CI 1.12–1.71) [67]. Vorapaxar is currently approved by the United States Food and Drug Administration as secondary prevention in patients with CAD or PAD. However, the marketing authorization was withdrawn in the European Union by the European Medicine Agency. In keeping with the targeting of thrombin, dual pathway inhibition with low-dose rivaroxaban was tested in the Cardiovascular Outcomes for People Using Anticoagulation Strategies (COMPASS) RCT in combination with aspirin in patients with stable atherosclerotic vascular disease. Rivaroxaban 2.5 mg plus aspirin vs. aspirin reduced MACE (HR 0.76; 95% CI 0.66–0.86), while there was a higher rate of major bleeding in the rivaroxaban plus aspirin arm (HR 1.70; 95% CI 1.40–2.05), there was no significant difference in intracranial or fatal bleeding between groups [68]. However, the benefits outweighed the risks, especially in patients with diabetes, renal dysfunction, HF or polyvascular disease [68]. In the LEPAD COMPASS substudy, rivaroxaban 2.5 mg plus aspirin prevented MACE prevention than aspirin alone (HR 0.74; 95% CI 0.58–0.92) and also prevented MALE (HR 0.55; 95% CI 0.35–0.85). ISTH major bleeding was increased in the combination therapy vs. aspirin alone (HR 1.69; 95% CI 1.18–2.40) with a numerical increase of fatal/critical organ bleeding (HR 1.56; 95% CI 0.78–3.39) [69]. Antithrombotic therapy in secondary prevention in LEPAD patients was assessed in RCTs after endovascular or surgical revascularization. The first trials on surgical revascularization assessed the efficacy of oral anticoagulation and aspirin or DAPT. Efficacy of oral anticoagulants compared with aspirin after infrainguinal bypass surgery (Dutch BOA) compared oral anticoagulation (i.e., phenprocoumon or acenocoumarol) vs. aspirin in patients who underwent infrainguinal grafting. No difference was detected between groups for the composite outcome of vascular death, MI, stroke or amputation (HR 0.89; 95% CI 0.75–1.06) or graft occlusion (HR 0.95; 95% CI 0.82–1.11) with an increased risk of major bleeding in patients treated with oral anticoagulation (HR 1.96; 95% CI 1.42–2.71) [47]. There was a similar finding from placebo-controlled clopidogrel and acetylsalicylic acid in bypass surgery for peripheral arterial disease (CASPAR) trial where aspirin plus clopidogrel vs. aspirin and placebo after BTK bypass graft did not show better outcomes considering index-graft occlusion or revascularization, above-ankle amputation of the affected limb, or death (HR 0.98; 95% CI 0.78–1.23), with increased total bleeding rate in the DAPT arm (16.7% vs. 7.1%, *p* < 0.001) without differences in severe bleeding (2.1% vs. 1.2%, *p* = NS) according to the GUSTO classification [48]. Cilostazol was investigated in LEPAD patients who underwent LER due to femoropopliteal lesions. In the “Cilostazol reduces restenosis after endovascular therapy in patients with femoropopliteal lesions” RCT, cilostazol was compared to ticlopidine in addition to aspirin in a DAPT strategy. Patency rates were higher in the cilostazol (73%) vs. ticlopidine arm (51%) at 36 months of follow-up (*p* = 0.013) with no differences in bleeding adverse events [49]. However, this was a small RCT with 127 patients enrolled and so not powered for clinical endpoints. Moreover, cilostazol is not currently used as an antithrombotic agent [19]. Cilostazol was assessed in LEPAD patients with moderate to severe claudication in a three-arm RCT compared to pentoxifylline and placebo. After 24 weeks, the improvement in the maximal walking distance by treadmill test was significantly greater in the cilostazol arm (107 ± 158 m) vs. pentoxifylline (64 ± 127 m with *p* < 0.001) and placebo (65 ± 135 m with *p* < 0.001) with more side effects as headache, diarrhea and palpitation in cilostazol group (*p* < 0.001) [70]. In subsequent metanalyses, cilostazol confirmed the improvement of maximal and pain-free walking distance of 15% in LEPAD patients affected by claudication and the main effect of cilostazol is represented by vasodilatation due to the increased level of cAMP [71]. However, the use of cilostazol is limited in patients with HF when associated with other phosphodiesterase 3 inhibitors and has known side effects such as headache, palpitations and tachycardia [4]. ACC/AHA GDMT suggests cilostazol with a class I recommendation as an effective therapy to improve symptoms and walking distance in LEPAD patients with claudication [4]. In the management of peripheral arterial interventions with mono or dual antiplatelet therapy (the MIRROR study), DAPT with aspirin plus clopidogrel vs. aspirin was assessed in patients who underwent endovascular LER. DAPT showed a lower rate of target lesion revascularization at 6 months (5% versus 8%, *p* = 0.04) but this benefit disappeared by 1 year (25% versus 32%, *p* = 0.35). Moreover, the bleeding complication rate at 6 months after LER was comparable among groups (2.5% vs. 5.0%, *p* = NS) [50]. The Edoxaban Plus Aspirin vs. Dual Antiplatelet Therapy in Endovascular Treatment of Patients With Peripheral Artery Disease (ePAD Trial) assessed edoxaban plus aspirin vs. standard DAPT with low-dose aspirin and clopidogrel. The trial was not powered for efficacy, and the risk of restenosis/reocclusion of femoropopliteal target lesion was not different between the two groups (HR 0.89; 95% CI 0.59–1.34). No significant excess in TIMI bleeding was observed with edoxaban, although the confidence interval was wide (HR 0.56; 95% CI 0.19–1.62) [51]. The efficacy of DPI after LER was assessed in the more recent Efficacy and Safety of Rivaroxaban in Reducing the Risk of Major Thrombotic Vascular Events in Subjects With Symptomatic Peripheral Artery Disease Undergoing Peripheral Revascularization Procedures of the Lower Extremities (VOYAGER-PAD) trial where PAD patients with LER were randomized to rivaroxaban 2.5 mg twice daily plus aspirin vs. placebo plus aspirin. Rivaroxaban plus aspirin showed a benefit in the primary efficacy outcome, including ALI, major amputation for vascular causes, MI, IS or death from CV causes (HR 0.85; 95% CI 0.76–0.96) [52]. No differences in TIMI major bleeding occurred between groups (HR 1.43; 95% CI 0.97–2.10), whereas ISTH major bleeding was higher in the rivaroxaban arm (HR 1.42; 95% CI 1.10–1.84) [52]. Subsequent analysis from the VOYAGER-PAD trial highlighted the benefits of rivaroxaban in reducing the first and subsequent events of the primary endpoint (HR 0.86; 95% CI 0.75–0.98) and vascular events (HR 0.86; 95% CI 0.79–0.95) [72]. In the VOYAGER-PAD, DPI benefit was observed in the primary efficacy outcome regardless of clopidogrel use (HR 0.85; 95% CI 0.71–1.01) or without clopidogrel (HR 0.86; 95% CI 0.73–1.01). Clopidogrel did not influence the main safety outcome of ISTH major bleeding with (HR 1.36; 95% CI 0.96–1.92) and without clopidogrel (HR 1.50; 95% CI 1.02–2.20) [73]. It was also noted in VOYAGER PAD that treatment with rivaroxaban reduced the risk for ALI (HR 0.67; 95% CI 0.55–0.82), particularly within the first month from randomization attesting an early benefit [74]. It is important to highlight that despite this tradeoff between efficacy and safety outcomes in VOYAGER PAD, when data are interpreted on an absolute rather than relative risk scale, the number of efficacy events prevented by rivaroxaban was exceedingly larger than the number of safety outcomes associated with a DPI strategy (181 events of efficacy outcomes prevented by rivaroxaban plus aspirin vs. 29 more safety events with the same strategy). Of interest, the median time from revascularization to randomization in the VOYAGER PAD trial was 5 days, suggesting that rivaroxaban should be started early after LER. It is noteworthy that LER in VOYAGER PAD was endovascular in approximately 65% of cases and surgical in the remaining 35%, therefore supporting the use of rivaroxaban irrespective of the type of treatment. Finally, femoro-popliteal revascularization in VOYAGER PAD represented about 90% of revascularization procedures, and therefore, this vascular district is probably key for the combination therapy of rivaroxaban and low-dose aspirin. A recent meta-analysis, including LEPAD patients from the COMPASS and VOYAGER trials, showed a favorable effect of rivaroxaban on the efficacy outcome of CV death, MI, IS, ALI or major amputation (HR 0.79; 95% CI 0.65–0.95) with an increased risk of ISTH major bleeding (HR 1.51; 95% CI 1.22–1.87). However, significant fatal or critical organ bleeding was similar between rivaroxaban and placebo (0.5% vs. 0.4% by year, *p* = NS) [75]. While the efficacy and safety of rivaroxaban were consistent regardless of DAPT use in VOYAGER PAD, few data are available on a direct comparison between DPI with aspirin and rivaroxaban vs. DAPT with aspirin and clopidogrel. A post-hoc analysis from VOYAGER PAD assessed the impact of aspirin plus rivaroxaban for a “CASPAR-like outcome”, including ALI, unplanned limb revascularization, amputation or CV death at 1 year in patients who underwent surgical LER according to CASPAR trial [48]. Considering 2185 patients of surgical LER group of VOYAGER PAD, rivaroxaban reduced the composite CASPAR-like endpoint at 1 year (HR 0.76; 95% CI 0.62–0.95) and increased ISTH major bleeding compared to placebo (HR 1.37; 95% CI 0.83–2.25) [76]. Although the aim of this analysis was not to compare the two trials, rivaroxaban showed benefit for a CASPAR-like outcome suggesting a role for thrombin inhibition in limb adverse events prevention even in surgical patients where the results in the CASPAR trial were neutral with DAPT [48]. An operative algorithm for antithrombotic management in LEPAD according to asymptomatic or secondary prevention patients is illustrated in Figure 2.

### 3.4. Antithrombotic Therapy in Peripheral Artery Disease Patients with Polyvascular Disease and Cardiovascular Comorbidities

Antithrombotic therapy in PAD patients affected by polyvascular disease and CV comorbidities may represent a challenge in daily practice. Considering patients with previous MI, the benefit of a DAPT strategy, including aspirin and ticagrelor, provided an absolute risk reduction of 4.1% for MACE and a 35% of MALE reduction counterbalanced by an increase of TIMI major bleeding (HR 1.32; 95% CI 0.41–4.29) in the PAD subgroup of PEGASUS-TIMI 54 RCT [63]. A secondary prevention strategy, including aspirin and low-dose rivaroxaban (DPI), confirmed a 26% MACE and 45% MALE reduction in an LEPAD COMPASS substudy with an increase of ISTH major bleeding vs. aspirin alone (HR 1.69; 95% CI 1.18–2.40). In this LEPAD COMPASS subanalysis, more than 50% of patients had a known history of CAD [69]. A different scenario is represented by PAD patients with recent PCI/acute coronary syndrome. Subanalyses of Dual Antiplatelet Therapy (DAPT) and Prolonging Dual Antiplatelet Treatment After Grading Stent-Induced Intimal Hyperplasia Study—PRODIGY RCTs demonstrated the efficacy of DAPT, even in PAD patients. In the DAPT trial, extended DAPT (with aspirin and either clopidogrel or prasugrel) reduced MI/stent thrombosis (HR 0,63; 95% CI 0.32–1.22) with a numerical increase of bleeding according to GUSTO classification (HR 1.82; 95% CI 0.87–3.83) among PAD patients [77]. The PRODIGY trial showed a significant MACE reduction comparing prolonged (24 months) vs. shorter DAPT (6 months) with aspirin and clopidogrel (HR 0.54; 95% CI 0.31–0.95) in PAD patients, particularly for those presenting with acute coronary syndrome (ACS) without significant differences in BARC ≥ 2 bleeding (HR 0.77; 95% CI 0.27–2.21) [60]. Therefore, in PAD patients with concomitant CAD, and particularly for those with recent ACS/PCI, GDMT supports 6–12 months of DAPT strategy with aspirin and either ticagrelor or prasugrel in patients without high bleeding risk, while a shorter DAPT (up to 6 months) with aspirin and clopidogrel in those with high bleeding risk [4,5]. Newer evidence is now available on the efficacy and safety of different duration of P2Y_12_ inhibitors in daily practice. Until last years, after DAPT discontinuation, the SAPT strategy included aspirin but new data suggest P2Y_12_ monotherapy as an effective antithrombotic strategy after short DAPT (up to 6 months) in ACS patients with high bleeding risk features [78]. DAPT with aspirin and clopidogrel up to 3 months is recommended by AHA/ASA guidelines after acute minor stroke due to carotid stenosis [8]. Atrial fibrillation (AF) is another common comorbidity among PAD patients due to several known common risk factor such as chronic kidney disease (CKD), type 2 diabetes mellitus, hypertension and advanced age [19]. In the subanalysis on PAD patients of Rivaroxaban versus Warfarin in Nonvalvular Atrial Fibrillation (ROCKET AF) RCT, rivaroxaban (15/20 mg daily) was compared to warfarin. Among patients with PAD, there were similar efficacy outcome rates considering stroke/systemic embolism for rivaroxaban vs. warfarin (HR 1.19; 95% CI 0.63–2.22) with significant interaction for major or clinically non-major relevant bleeding in PAD patients (HR 1.40; 95% CI 1.06–1.86) compared to those without PAD (HR 1.03; 95% CI 0.95–1.11), p-interaction 0.037 [79]. However, the difference in major bleeding rates between rivaroxaban and vitamin K antagonists (VKAs) was not confirmed in subsequent studies involving PAD patients (HR 1.13; 95% CI 0.84–1.52) [80]. The efficacy in AF prevention by direct oral anticoagulants compared to warfarin in PAD patients was confirmed by subanalysis of The Apixaban for Reduction in Stroke and Other Thromboembolic Events in Atrial Fibrillation (ARISTOTLE) trial. The risk of stroke/systemic embolism was similar for apixaban vs. warfarin in patients with PAD (HR 0.63; 95% CI 0.32–1.25) with no differences in major or clinically non-major relevant bleeding (HR 1.05; 95% CI 0.69–1.58) [81]. More recently, the safety and effectiveness of edoxaban vs. standard practice of dosing with warfarin in patients with atrial fibrillation (ENGAGE AF-TIMI 48) trial, evaluated edoxaban vs. warfarin on systemic embolism and major bleeding in AF patients with concomitant PAD. Even in PAD patients, edoxaban was not inferior compared to warfarin considering systemic embolism (HR 1.16; 95% CI 0.42–3.20) and for ISTH major bleeding (HR 0.96; 95% CI 0.54–1.70) [82]. Hence, in PAD patients with concomitant AF, GDMT recommends oral anticoagulation [4,5,19]. In case of endovascular LER with stenting or CAS, adding SAPT to oral anticoagulation for 1 month may represent an option in AF patients without high bleeding risk [19]. Patients with end-stage CKD/dialysis are often affected by ASCVD, including PAD and therefore are considered a very high CV risk subgroup patients for MACE, MALE and bleeding adverse events [83]. Moreover, the presence of end-stage CKD/dialysis dependence in patients is not an isolated disease and often coexists with PAD and major tissue loss (i.e., Rutherford category 6) [83]. These patients are usually excluded from RCTs on antithrombotic therapy, even in a PAD setting. However, new data on the safety and efficacy of DOACs in CKD are available from an RCT (Valkyrie study), which compared rivaroxaban vs. VKA in dialysis patients with AF. There, 132 patients were randomized among three groups, VKA, rivaroxaban (10 mg daily) or rivaroxaban plus vitamin K2 for 18 months. Compared to VKA, rivaroxaban reduced the primary efficacy endpoint of fatal and non-fatal CV events (HR 0.41; 95% CI 0.25–0.68) in the rivaroxaban group and HR 0.34 (95% CI 0.19–0.61) in the rivaroxaban and vitamin K2 group. Symptomatic limb ischemia occurred more frequently in VKA (45%) than in rivaroxaban groups (22%), *p* = 0.02. There were fewer life-threatening and major bleeding adverse events in the rivaroxaban arm (HR 0.39; 95% CI 0.17–0.90) and in the rivaroxaban plus vitamin K2 arm (HR 0.48; 95% CI 0.22–1.08) compared to the VKA arm [84]. Further data from larger RCTs are needed to determine the optimal anticoagulation strategy in dialysis patients with AF. Moreover, two recent phase 2 RCTs on factor XI inhibitors (IONIS-FXI_Rx_ and Xisomab 3G3) were conducted in dialysis patients with promising results on FXI inhibition with no evidence of impaired major bleeding rates compared to placebo [85]. Other ongoing RCTs will assess the efficacy and safety of factor XI inhibitors in a larger cohort of dialysis patients [85]. Hence, established safety and efficacy data of newer antithrombotic therapy in PAD patients, such as DPI, are not available in patients with end-stage CKD/dialysis and related complications. Despite the fact that having end-stage CKD/dialysis per se is not an exclusion criterion for aspirin and P2Y_12_, such as clopidogrel and ticagrelor, there are few available data, particularly on safety profiles in PAD patients. Balancing thrombo/hemorrhagic risk, an SAPT strategy with aspirin or clopidogrel is recommended according to Kidney Disease Improving Global Outcomes guidelines in patients with end-stage renal disease/dialysis with concomitant ASCVD [86].

## 4. Conclusions

PAD patients are at increased risk of MACE and MALE events that can be reduced by antithrombotic therapy. We have provided an overview of current evidence in different clinical settings in PAD and propose an algorithm for antithrombotic therapy management in daily practice. In patients undergoing revascularization, evidence supports more aggressive antithrombotic therapy, specifically, dual pathway inhibition after LER, irrespective of the type of intervention. The optimal management of patients undergoing revascularization for carotid and abdominal aortic disease is less clear. The development of newer antithrombotic strategies (i.e., factor XIa inhibition) may play an important role in the future.

## Figures and Tables

**Table 1:** Key antithrombotic trials in a primary population with peripheral artery disease.

Study	Enrolled Patients	Population	Treatment	Follow-Up	Primary Endpoint	
					Efficacy	Safety
Carotid Artery Disease						
ESPRIT Trial [24]	1068	Recent TIA or minor stroke (within 6 months)	Oral anticoagulation (phenprocoumon, warfarin or acenocoumarol) vs. Aspirin	4.6 years	No difference in the composite outcome of all-cause death, non-fatal stroke, non-fatal MI (HR 1.02; 95% CI 0.77–1.35)	Increased major bleeding (HR 2.56; 95% CI 1.48–4.43)
CARESS Trial [26]	107	Symptomatic ≥50% carotid stenosis	DAPT (Aspirin + Clopidogrel) vs. Aspirin	7 days	Reduction in the risk of asymptomatic embolization (RR 39.8%; 95% CI 13.8–58.0)	Bleeding adverse events 3.9% vs. 1.8%, *p* = NS
CLAIR Trial [27]	100	Acute IS or TIA	DAPT (Aspirin + Clopidogrel) vs. Aspirin	7 days	Reduction of microembolic signals (RR 42.4%; 95% CI 4.6–65.2)	No difference in any bleeding complications
CHANCE Trial [28]	5170	Minor IS or high-risk TIA	DAPT (Aspirin + Clopidogrel) vs. Aspirin + Placebo	90 days	Reduction of stroke rate in the DAPT group (HR 0.68; 95% CI 0.57–0.81)	No difference in bleeding complications (HR, 1.41; 95% CI 0.95–2.10)
POINT Trial [29]	4881	Minor IS or high-risk TIA	DAPT (Aspirin + Clopidogrel) vs. Aspirin	90 days	Reduction of major ischemic events (IS, MI and death due to ischemic vascular events) (HR 0.75; 95% CI 0.59–0.95)	Increased hemorrhagic complications (HR 2.32; 95% CI 1.10–4.87)
Dual Antiplatelet therapy using Cilostazol for Secondary Prevention in Patients with high-risk ischemic stroke in Japan [32]	1884	High-risk non-cardioembolic IS	Aspirin/Clopidogrel + Cilostazol vs. Aspirin or Clopidogrel	1.4 years	Reduction of IS in the DAPT group (HR 0.49; 95% CI 0.31–0.76)	No difference in severe or life-threatening bleeding (HR 0.66; 95% CI 0.27–1.60)
SOCRATES Trial [33]	13,199	Non-severe IS or high-risk TIA	Ticagrelor vs. Aspirin	90 days	No difference in the composite outcome of stroke, MI or death (HR 0.89; 95% CI 0.87–1.01)	No difference in major bleeding complications (HR 0.83; 95% CI 0.52–1.34)
THALES Trial [35]	11,016	Mild-to-moderate acute noncardioembolic IS or TIA	DAPT (Aspirin + Ticagrelor) vs. Aspirin	30 days	Reduction of composite of stroke or death in the DAPT group (HR 0.83; 95% CI 0.71–0.96)	Increased severe bleeding (HR 3.99; 95% CI 1.74–9.14)
The Benefits of Combined Antiplatelet Treatment in Carotid Artery Stenting [36]	47	Patients undergoing carotid artery stenting	DAPT (Aspirin + Clopidogrel) vs. Aspirin + 24-h heparin	30 days	Neurological events (amaurosis fugax, TIA and all stroke) 0% vs. 25%	No difference in major bleeding 9% vs. 17%, *p* = NS
Dual Antiplatelet Regime Versus Acetyl-acetic Acid for Carotid Artery Stenting [37]	100	Patients undergoing carotid artery stenting	DAPT (Aspirin 325 mg + Ticlopidine) vs. Aspirin 325 mg + 24-h heparin	30 days	Reduction of minor IS/TIA in the DAPT group, 2% vs. 16%, *p* < 0.05	No difference in major bleeding
ACE RCT [38]	2849	Patients undergoing carotid endarterectomy	Aspirin 81–325 mg vs. Aspirin 650–1300 mg	90 days	Lower rate of IS, MI and death in low-dose group 6.2% vs. 8.4%, *p* = 0.03	Increased hemorrhagic stroke in high dose group (RR, 1.68; 95% CI 0.77–3.68)
PACIFIC Stroke Trial [41]	1808	Acute non-cardioembolic IS	Asundexian 10 mg vs. Asundexian 20 mg vs. Asundexian 50 mg vs. Placebo	26 weeks	No differences in IS and incident covert brain infarct detected by MRI (HR 0.99; 95% CI, 0.79–1.24) 10 mg (HR 1.15; 95% CI 0.93–1.43) 20 mg (HR 1.06; 95% CI 0.85–1.32) 50 mg	No difference in major or clinically relevant non-major bleeding (HR 1.57; 95% CI 0.91–2.71)
AXIOMATIC-SPP [42]	2366	Acute non-lacunar IS	Milvexian 25 mg vs. Milvexian 50 mg vs. Milvexian 100 mg vs. Milvexian 200 mg vs. Placebo	90 days	No differences in IS and incident covert brain infarct detected by MRI 25 mg, 16.2% and 18.5% 50 mg, 14.1% 100 mg, 14.7% 200 mg, 16.4% Placebo, 16.6%	No differences in rates of BARC 3 or 5 25 mg, 0.6% 50 mg, 1.5% 100 mg, 1.6% 200 mg, 1.5% Placebo, 0.6%
**Abdominal Aortic Disease**						
TicAAA Trial [43]	144	Patients with AAA and with a maximum aortic diameter 35–49 mm	Ticagrelor vs. Placebo	12 months	No differences were found in AAA volume increase assessed by MRI (HR 1.013; 95% CI 0.993–1.034)	Increased bleeding events rate in ticagrelor group (33% vs. 11%, *p* = 0.002)
**Lower-extremity PAD**						
POPADAD Trial [44]	1276	Patients with diabetes with ABI < 0.99	Aspirin vs. Placebo	6.7 years	No difference in the composite of death due to CAD or stroke, non-fatal MI or stroke, or amputation (HR 0.98; 95% CI 0.76–1.26)	No difference in gastrointestinal bleeding (HR 0.90; 95% CI 0.53–1.52)
AAA Trial [45]	3350	General population with ABI ≤ 0.95	Aspirin vs. Placebo	8.2 years	No difference in the composite of fatal or non-fatal coronary event, stroke, or revascularization (HR 1.03; 95% CI 0.84–1.27)	Increased major bleeding (HR 1.71;95% CI 0.99–2.97)
WAVE Trial [25]	2161	PAD	Antiplatelet therapy (aspirin, ticlopidine or clopidogrel) + Oral anticoagulation (warfarin or acenocoumarol) vs. Antiplatelet therapy alone	35 months	No difference in the composite outcome of all-cause death, stroke and MI (RR 0.92; 95% CI 0.73–1.16)	Increased life-threatening or moderate bleeding (RR 3.21; 95% CI 2.02–5.08)
EUCLID Trial [46]	13,885	Symptomatic LEPAD	Ticagrelor vs. Clopidogrel	30 months	Ticagrelor not superior to clopidogrel for MACE reduction (HR 1.02; 95% CI 0.92–1.13)	No increase in bleeding (HR 1.1; 95% CI 0.84–1.43)
Dutch BOA [47]	2690	Patients with LEPAD after infrainguinal arterial grafting	Oral anticoagulant (phenprocoumon or acenocoumarol; coumarin derivatives) vs. aspirin equivalent	21 months	No difference in graft occlusion (HR 0.95; 95% CI, 0.82–1.11) No difference in the composite of vascular mortality, MI, stroke, or amputation (HR 0.89; 95% CI 0.75–1.06)	Increase in severe bleeding (HR 1.96; 95% CI 1.42–2.71)
CASPAR Trial [48]	851	Patients with LEPAD undergoing below-knee bypass grafting	Aspirin + Clopidogrel vs. Aspirin + Placebo	24 months	No reduction in the composite of graft occlusion, revascularization, major amputation, or death (HR 0.98; 95% CI 0.78–1.23)	No difference in severe bleeding (2.1% vs. 1.2%)
Cilostazol reduces restenosis after endovascular therapy in patients with femoropopliteal lesions [49]	127	Patients with LEPAD after endovascular LER	Aspirin + Cilostazol vs. Aspirin + Ticlopidine	36 months	Higher patency lesion rates at 12, 24, 36 months in Cilostazol group (87%, 82%, 73%) vs. Ticlopidine group (65%, 60%, 51%), *p* = 0.013	No difference in bleeding *p* = 0.72
MIRROR Study [50]	80	Patients with LEPAD undergoing endovascular LER	Clopidogrel + Aspirin vs. Aspirin + Placebo	6 months	Decreased risk of target lesion revascularization (5% vs. 8%, *p* = 0.04) at 6 months but no difference at 1 year (25% vs. 32%, *p* = 0.35)	No increase in bleeding (2.5% vs. 5%, *p* = 0.56)
ePAD Trial [51]	203	Patients with LEPAD after endovascular LER	Edoxaban + Aspirin vs. Aspirin + Clopidogrel	3 months	No difference in restenosis or reocclusion of femoropopliteal targets (HR 0.89; 95% CI 0.59–1.34)	No difference in bleeding (RR 0.56; 95% CI 0.19–1.62)
VOYAGER-PAD Trial [52]	6564	Patients with LEPAD after LER	Aspirin + Rivaroxaban vs. Aspirin + Placebo	3 years	Composite of reduction of MACE and MALE (HR 0.85; 95% CI 0.76–0.96)	No difference in TIMI major bleeding (HR 1.43; 95% CI 0.97–2.10) increase in ISTH major bleeding (HR 1.42; 95% CI 1.10–1.84)

HR = hazard ratio; CI = confidence interval; NS = not significant; RR = risk ratio; TIA = transient ischemic attack; DAPT = dual antiplatelet therapy; IS = ischemic stroke; MI = myocardial infarction; AAA = abdominal aortic aneurysm; MRI = magnetic resonance imaging; ABI = ankle brachial index; PAD = peripheral artery disease; CAD = coronary artery disease; MACE = major adverse cardiovascular events; MALE = male adverse limb events; TIMI = thrombolysis in myocardial infarction; ISTH = International Society on Thrombosis and Hemostasis.

## Data Availability

All data underlying this article will be shared on reasonable request to the corresponding author.
